# Anti-inflammatory effects and beneficial effects of the feed additive *Urtica cannabina* L. in zebrafish

**DOI:** 10.1371/journal.pone.0307269

**Published:** 2024-07-17

**Authors:** Wuyun Liu, Huarong Yu, D. Gurbazar, D. Rinchindorj, Wei Kang, Chelimuge Qi, Hongsong Chen, Xu Chang, Huan You, Yongmei Han, Zhigang Li, Ahmed R. G., Wu Dong

**Affiliations:** 1 Key Laboratory of Ecological Agriculture in Horqin Sandy Land, State Ethnic Affairs Commission, Wuhan, China; 2 College of Agriculture, Inner Mongolia Minzu University, Tongliao, Inner Mongolia, China; 3 Mongolian University of Life Sciences, School of Animal science & Biotechnology, Ulaanbaatar, Mongolia; 4 Inner Mongolia Key Laboratory of Toxicant Monitoring and Toxicology, College of Animal Science and Technology, Inner Mongolia Minzu University, Tongliao, Inner Mongolia, China; 5 Tongliao Animal Husbandry Development Center, Tongliao, Inner Mongolia, China; 6 Faculty of Science, Zoology Department, Division of Anatomy and Embryology, Beni-Suef University, Beni-Suef, Egypt; Universitat Politècnica de València, SPAIN

## Abstract

*Urtica cannabina* L. (UL) has been used clinically for centuries because of its anti-inflammatory properties. This study aimed to investigate the underlying mechanisms and anti-inflammatory effects of different UL concentrations in zebrafish. To elucidate UL’s anti-inflammatory properties, two inflammation zebrafish models were designed 1) by severing the zebrafish’s caudal fin to assess the repairing effect of UL on the tail inflammation, and 2) by inducing lipopolysaccharides (LPS)-mediated intestinal inflammation to assess the protective and reparative effects of UL on intestinal inflammation at the histological and genetic levels. Furthermore, the effect of UL on the LPS-induced intestinal flora changes was also assessed. After caudal fin resection, a scar formed on the tail of the zebrafish, and the area of the caudal fin increased by 1.30 times as much as that of the control group (*P* < 0.01). Moreover, this tail scar was alleviated after 10 mg/g UL supplementation but not after 30 mg/g UL dose. LPS decreased the feed intake and body weight of the zebrafish; however, these effects were reversed after 10 and 30 mg/g doses of UL. In addition, the LPS treatment also reduced the intestinal goblet cells by 49% in the zebrafish when compared with the control, which was significantly restored after 10 and 30 mg/g UL treatments. At the genetics level, the expression of the pro-inflammatory cytokine genes (*TNF-α*, *IL6*, and *IL8*) showed that 10 and 30 mg/g UL doses could rescue LPS-induced expression. The gut microbiota analysis revealed changes in the abundance of four major bacterial phyla in the 10 and 30 mg/g UL-treated groups, with an increased probiotic Bacteroidota and decreased pathogenic bacteria. These results indicate that UL strongly inhibits inflammation caused by caudal fin removal and LPS-induced inflammatory changes in the zebrafish intensity, suggesting that UL is a feed additive that could be developed to improve resistance to inflammation in livestock.

## Introduction

Natural plants have medicinal properties and therefore, have been widely studied for their disease-preventive effects. Furthermore, natural plants with anti-inflammatory properties have been extensively studied. Ahmadifar *et al*. reported that ginger powder (especially 3%) elevated the expression of antioxidant and immune system-related genes and enhanced immune function in zebrafish [*Danio rerio* (Hamiltom,1822)] [1]. Furthermore, Safari *et al*. added myrtle to the zebrafish diet and indicated elevated expression of antioxidant-related genes and improved mucosal immune responses (lysozyme, total immunoglobulin and protease activities) [2]. In this study, zebrafish were used as a model animal to investigate the anti-inflammatory effects of nettles (*Urtica*).

Nettles are a group of perennial herbs that are produced globally [3] and comprises 31 compounds, including 8 flavonoids, 14 phenolic compounds, 8 phenylpropanoid classes, and 1 terpenoid [4]. Its applications in food and medicine has become a research hotspot. For example, studies on the effects of Urtica dioica L. powder on nutrient bioavailability have been performed [5,6], which revealed that the extract had antibacterial activity [7] and various medicinal properties [8]. In this genus, *Urtica cannabina* L. (UL) is widely used in the preparation of herbal medicine and has a long history as a medicinal plant in China [9]. Currently, it is predominantly used for the anti-inflammatory treatment of arthritis and contains several biologically active substances, such as lignans, flavonoids, and other phenolic compounds [10]. Recently, this herb was found to be a beneficial additive to animal feed. It was observed that the addition of UL, increased the feed digestibility and improved skeletal and muscle growth in Mongolian bulls (*Bos taurus Linnaeus*) [11]. Moreover, UL also significantly improved the feed utilization rate in goats (*Capra hircus Linnaeus*) [12], which may be related to its antioxidant effect and the improvement of liver functions [13]. In lamb (*Ovis aries Linnaeus*), UL supplementation not only improved feed digestibility, but also gut health [14], general physiology, and muscle fatty acid composition [15].

Lipopolysaccharides (LPSs) are used to establish zebrafish inflammation models, which can be employed to assess the anti-inflammatory effect of glucocorticoids, explore the mechanism of drugs, and develop new glucocorticoid drugs [16]. For example, Zhang *et al*. utilized the LPS zebrafish inflammation model to examine the anti-inflammatory activity of the total indolealkylamines (extracted from toad skin) [17] and indicated that *Boswellia serrata* Roxb. can act as a potent redox scavenger against LPS-induced inflammation in larval zebrafish [18]. Unlike the antagonistic drugs that were mentioned above, Polygoni Multiflori Radix (RPM) has no antagonistic effect on the LPS-induced inflammatory changes. Instead, LPSs increases the absorption of potentially toxic RPM’s components, thereby inhibiting the metabolism [19]. Research on the anti-inflammatory and beneficial effects of UL is still lacking, therefore, this study aimed to explore the anti-inflammatory effects of UL on zebrafish inflammation models.

Zebrafish are a common biological model in molecular biology, pharmacology, and toxicology research [20] and are also widely used to study mechanisms of growth and development. Due to their unique advantages in biology, genomics, and genetics as well as highly conserved signaling pathways, they are ideal model organism for disease research [21]. Zebrafish have been used as a fish model for studying intestinal inflammation induced by a plant-based diet [22]. Furthermore, it has been observed that tannins can improve intestinal inflammation in zebrafish with a terrestrial plant-based diet [23]. Therefore, this study established a zebrafish inflammation model using LPSs to induce intestinal and liver inflammation to evaluate the anti-inflammatory and reparative effects of UL as a feed additive. Moreover, the safety of UL as a feed was assessed in terms of morphology, histology and intestinal flora. In addition, the anti-inflammatory effect of UL on tail inflammation induced by the severing of the caudal fin and intestinal inflammatory response caused by LPS exposure was investigated.

## Materials and methods

### Animals and treatment

The zebrafish (AB strain) were maintained in a re-circulating water culture system (Aisheng Technology, Beijing, China) at 28 ± 1°C, 7.2–7.8 pH with 440–640 μS conductivity, and a 14/10 h light/dark. The zebrafish were fed with commercial feed twice daily (Beijing Botai Hongda Biotechnology, Beijing, China). The nutritional value of the feed is provided in Table 1. Four-month-old male and female fish were used in the experiments. Tricaine Methanesulfonate (5–250 mg/L; MS-222, Sigma-Aldrich, St. Louis, MO, USA) was utilized as an anesthetic for the zebrafish [24]. Zebrafish were euthanized by unbuffered MS222 solution (250 mg/L) and immersion in an ice-water bath, their liver were harvested, rapidly placed in a 2 mL cryotube and then in liquid nitrogen for transcriptome sequencing and mRNA transcription analysis. The intestine of the zebrafish was dissected, rinsed with physiological saline, and then its contents were squeezed out using a sterile cotton tip before storage at -80°C for 16S rRNA sequencing [25]. The adult care and reproductive techniques were noninvasive and approved by the Inner Mongolia Minzu University for the Nationalities Institutional Animal Care and Use Committee (NM-LL-2023-08-14-01).

**Table 1:** The nutritional value of the feed and *Urtica cannabina* L. (UL).

Item/%	UL	Fish feed
Dry matter/DM	96.81	97.31
Crude protein/CP	16.09	49.0
Crude fat/EE	3.05	5.0
Coarse Ash /Ash	17.24	3.0
Neutral detergent fiber/NDF	40.89	3.0
Calcium/Ca	0.58	5.0
Phosphorus/P	0.90	1.5

### Experimental diet preparation

The UL specimen was harvested from Hexigten Banner (42°15’23.50’’N, 118°52’58.14’’E), Chifeng City, Inner Mongolia Autonomous Region and identified according to Mehrabi *et al*. method [26]. The UL leaves were rinsed with deionized water, air dried, and then pulverized into a fine powder with a particle size of 380 μm. Then, 0.05 g of the powder was mixed with 4.95 g of commercial fish feed after the addition of 5 mL of distilled water to create a 10 mg/g UL feed. A 30 mg/g UL feed was similarly made by mixing 0.15 g of UL and 4.85 g of zebrafish feed. The resultant mixtures were air-dried in the laboratory and passed through a 100 μm filter to create pellets that were a suitable size for the zebrafish. For feeding the controls, same protocol was followed but UL powder was not added. The nutritional value of the UL is presented in Table 1.

### Zebrafish feeding and exposure experiments

**Fig 1:**
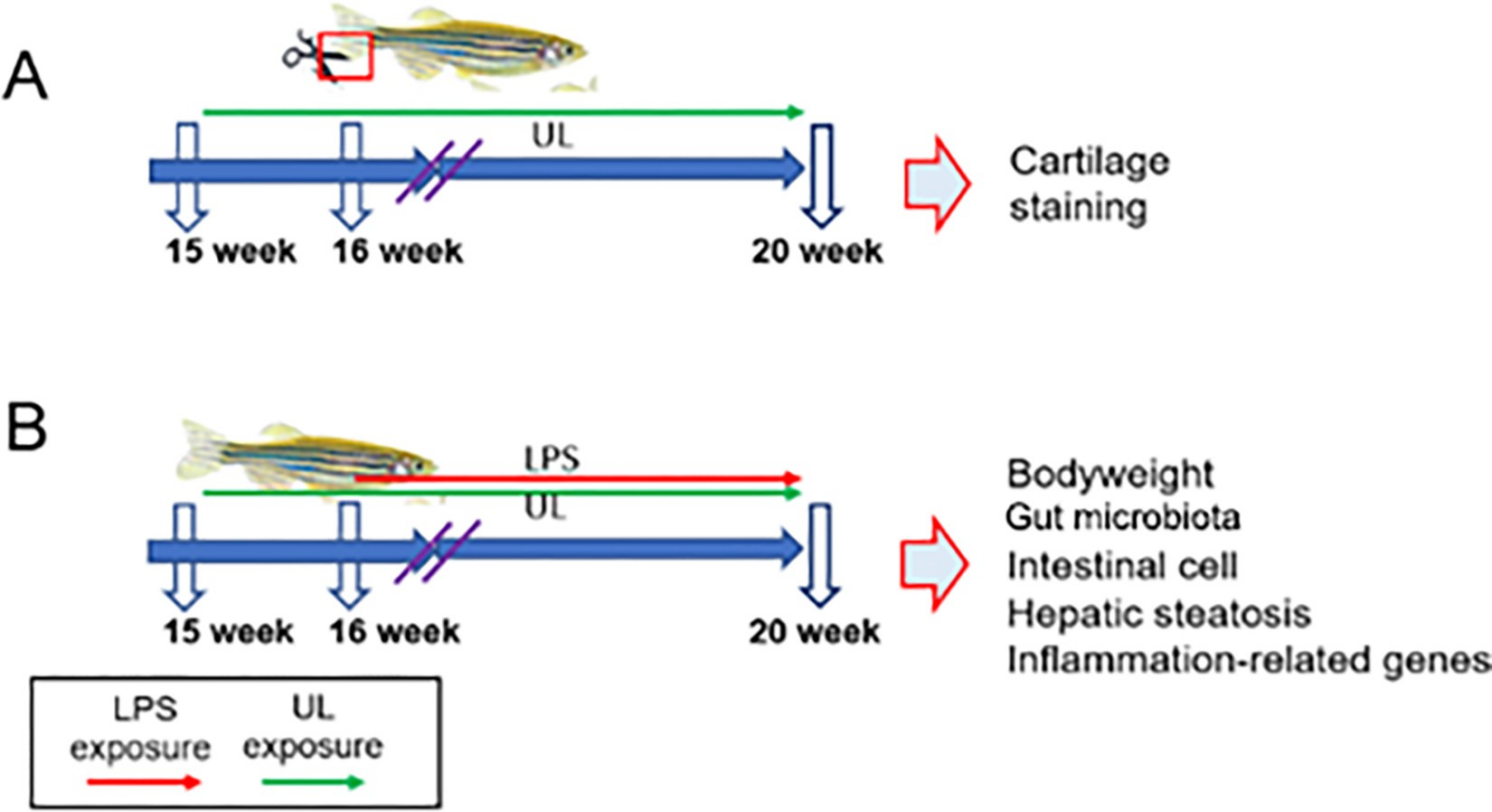
Schematic diagram of the experimental design. Abbreviations: LPS: Lipopolysaccharides, UL: *Urtica cannabina* L. (A): UL supplemented diets were fed from week 15 to 20, caudal fin resection was performed at week 16, and zebrafish larvae were collected for cartilage and bone staining at week 20. (B): The feed with UL alone was fed until week 15, LPS was added at week 16, and the body weight, gut microbiota, hepatic steatosis, and gene expression were monitored at week 20.

The 13-week-old adult zebras were randomly selected and transferred to experimental tanks at a density of five fish (550 mg) per liter [27,28]. After two-week acclimation period (at week 15), the experiment was started. The fish in each tank were fed twice a day with feed supplemented with UL (0, 10, or 30 mg/g) [29]. Two experiments were carried out concurrently. In the first experiment (Fig 1A), the fish were exposed to experimental diets for 2 weeks and then their caudal fins were clipped (5 mg/mL MS-222 was added in the culture solution for pain relief). Then, caudal fin regeneration was observed for 4 weeks, and the fish were removed for Alcian Blue and Alizarin Red double staining to visualize the regrowth of the caudal fin. Image J software (National Institutes of Health version 1.49) was used to measure the area of the zebrafish caudal fin. In the second experiment (Fig 1B), the fish were exposed to experimental diets for 2 weeks and then exposed to LPSs from *Escherichia coli* (Migula 1895) (purity ≥ 99%; Sigma-Aldrich). This study included 6 treatment groups, the control (UL: 0 mg/g), 10 mg/g UL, 30 mg/g UL, LPS, LPS + 10 mg/g UL, and LPS + 30 mg/g UL groups. The LPS and UL + LPS groups received drops of 1 mL LPS at a concentration of 50 μg/mL every 5 d. Each treatment was repeated in triplicate with 10 fish per replicate. At the end of the treatments, the body weight, gut microbiota composition, intestinal histology, and expression of the inflammation-related genes were measured.

### Alcian Blue and Alizarin Red staining

The zebrafish caudal fins were fixed overnight in 4% paraformaldehyde and stained with Alcian Blue [0.1 g Alcian Blue in 70 mL ethanol and 30 mL glacial acetic acid (Solarbio, Beijing, China)] overnight at room temperature. The next day, the caudal fins were washed with 95, 90, 80, 70, 50, and 20% ethanol and phosphate-buffered saline (PBS). The fins were then digested using 10 mg/mL trypsin in 30% saturated aqueous sodium borate overnight and then bleached with 3% hydrogen peroxide for 1 h until the sample was transparent. The Alizarin Red solution [0.4 mL saturated Alizarin Red S in ethanol diluted in 10 mL 0.5% KOH (Solarbio, Beijing, China)] was used to stain the fins until the bony structures turned red. The samples were then examined under a stereomicroscope (Leica, Germany).

### Weight measurement and intake calculation

The weight of the zebrafish was measured on days 1 and 30. To calculate the daily weight gain of the zebrafish; weight of the zebrafish at 1 d was subtracted from that on 30 d and then divided by the weight at 30 d. The formula for average daily feed intake = total feed intake/days of feeding/number of fish.

### Tissue sectioning and staining

The zebrafish intestines were collected, fixed in 4% paraformaldehyde overnight at 4°C, and then encapsulated in paraffin after a series of ethanol and xylene treatments. The tissues were then cut into 5 μm sections and stained with hematoxylin-eosin and periodic acid-Schiff (PAS) [25,30].

### Total RNA extraction and quantitative real-time polymerase chain reaction

The total RNA of the adult zebrafish liver tissue was extracted using TRIzol (Invitrogen, Carlsbad, CA, USA). The RNA sample’s purity was determined using a NanoDrop ND-2000 spectrophotometer (Thermo Fisher Scientific, Waltham, MA, USA). The RNA sample with a 260/280 nm ratio of > 1.8 value was employed for further analysis. A High-Capacity cDNA Reverse Transcription Kit (Applied Biosystems Inc., Foster City, CA, USA) was used to reverse transcribe the RNA into complementary DNA (cDNA). Then, a quantitative real-time polymerase chain reaction was carried out as described by Dong *et al*. [30] to determine the gene expression. Genes related to inflammation (*TNF-α*, *IL-6*, and *IL-8*) were selected and studied, while 18S was used as an internal reference. The primer sequences used are presented in Table 2. The relative gene expression was calculated using the 2^-ΔΔCT^ method.

**Table 2:** Primer sequences for the genes that were investigated in this study.

Gene	Primer Sequence (5’ to 3’)	Gene numbers	Length (bp)
*TNF-α*	F:GCTGGATCTTCAAAGTCGGGTGTA R:TGTGAGTCTCAGCACACTTCCATC	NM_212859.2	138
*IL-8*	F:TGGCATTTCTGACCATCATTG R:TCTTCTTAACCCATGGAGCA	XM_009306855.3	222
*IL-6*	F:GGTGAGAGACGGAGAGATGGAT R:CACGCTGGAGAAGTTGAACAG	NM_001261449.1	109
*18S*	F:TCGCTAGTTGGCATCGTTTATG R:CGGAGGTTCGAAGACGATCA	NR_145818.1	241

### Sequencing of the gut microflora diversity

At the end of the first experiment, six zebrafish from each treatment were euthanized and their intestines were dissected, rinsed with PBS, cut open and swabbed with a sterile cotton swab. The genomic DNA from the samples was extracted using the Fast DNA SPIN Kit (MP Biomedicals, Santa Ana, CA, USA). An amplicon of 16S ribosomal RNA (rRNA) was generated using polymerase chain reaction (PCR) and the upstream primer 338F (5′-ACTCCTACGGGAGGCAGCAGCAG-3′) and the downstream primer 806R (5′-GGACTACHVGGGGTWTCTAAT-3′) for the V3–V4 region. The PCR product was purified by gel electrophoresis and quantified for the construction of a sequencing library. The sequencing libraries were generated using the TruSeq DNA PCR-Free Sample Preparation Kit (Illumina, San Diego, CA, USA), per the manufacturer’s recommendations, and the index codes were added. The library quality was assessed using the Qubit 2.0 Fluorometer (Thermo Fisher Scientific) and Agilent Bioanalyzer 2100 system (Agilent Technologies Inc., Santa Clara, CA, USA). The library was then sequenced on an Illumina NovaSeq platform (Illumina), and 250 bp paired-end reads were generated. The normalized operational taxonomic unit (OTU) sequences were aligned against the SILVA database and clustered into different taxonomic levels (phyla, families, and genera) with a cutoff of 97% similarity.

### Statistical analyses

All the data are expressed as the mean ± standard error. The endpoints were compared using a one-way analysis of variance and the Newman-keels test, and *p* < 0.05 was considered statistically significant. A permutational multivariate analysis of variance test with Bonferroni correction was used to compare the differences in the phylogenetic structure among the three groups to acquire the 16S microbiota data [31]. Furthermore, the species diversity indices (Simpson, Shannon, Chao1, and ACE) of the three treatments were computed using QIIME (Version 1.7.0). Moreover, the principal coordinate analysis was performed to visualize the similarity among the bacterial community composition in the three treatments using R (Version 2.15.3) [25].

## Results

### Effect of *Urtica cannabina* L. on zebrafish caudal fin regeneration

**Fig 2:**
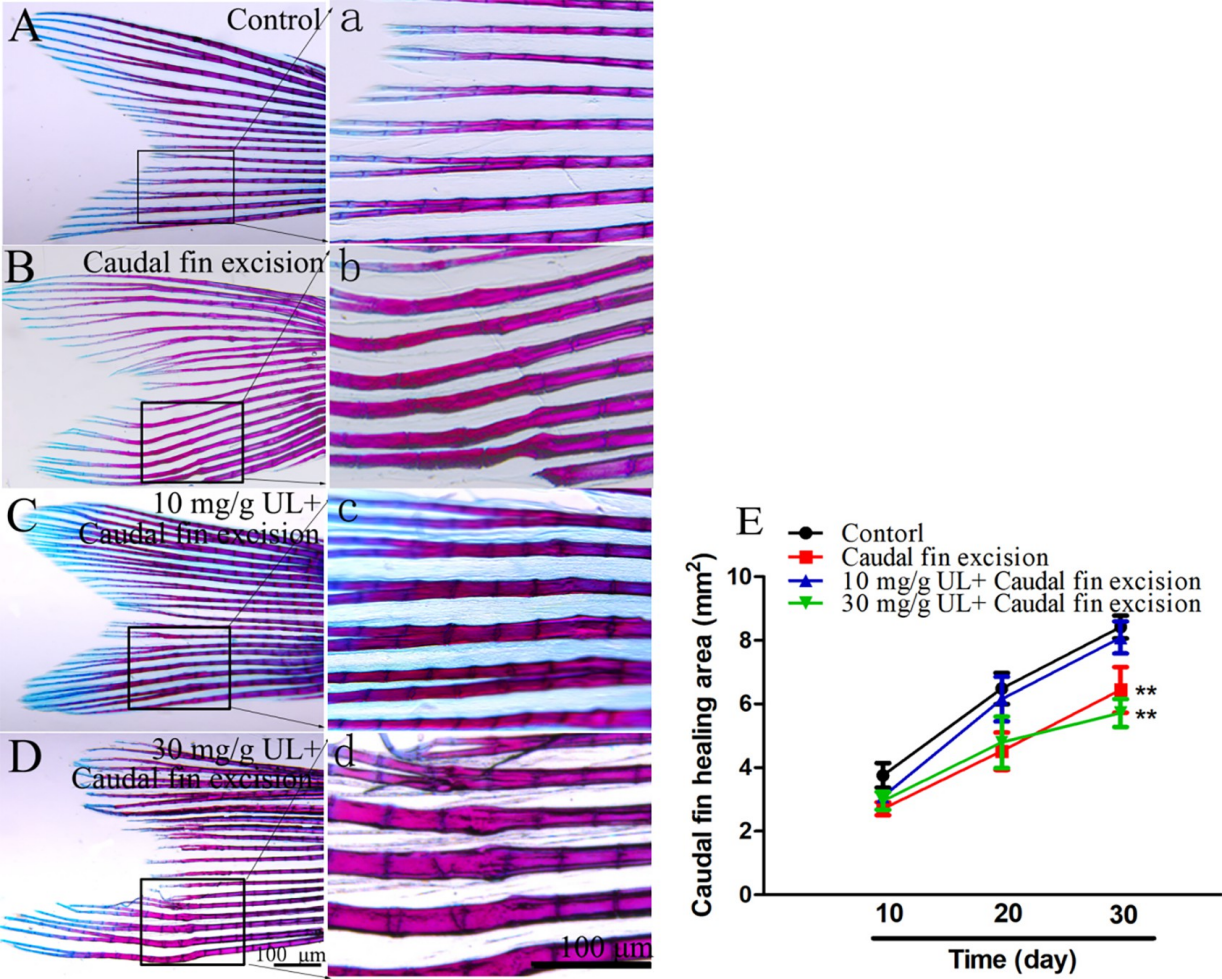
Effect of *Urtca cannabina* L. (UL) on wound recovery and bone regeneration in the caudal fin of the zebrafish. The area of regrowth in the caudal fin for each concentration group was compared. (A): Negative control group (no excision), (B): Positive control group (caudal fin excision but no UL feeding). (C): 10 mg/g and (D): 30 mg/g UL feeding after caudal fin excision; (E): Changes in the caudal fin area over time. The asterisks (*) indicate significant differences between the exposed and control groups (*p < 0.05; **p < 0.01; ***p < 0.001); the data are presented as mean ± the standard deviation (n = 10). Scale bar = 100 μm.

The caudal fins of the zebrafish exhibited regrowth in all the groups. The regrowth was the fastest for the 10 mg/g UL group, and complete regrowth was observed 20 d after clipping (Fig 2E). Furthermore, the caudal fin of the control group regrew significantly slower than that of the 10 mg/g UL group and was similar to that of the 30 mg/g UL group. A minor deformity was identified in the regrowth tissue of the caudal fin. The location of the clipping was visible even after 30 d (Fig 2A–2D). In addition, the new caudal fin growth indicated minor twisting in the control and 30 mg/g UL groups (Fig 2B and 2D); however, this was not observed in the 10 mg/g UL group (Fig 2C).

### Inhibitory effect of *Urtica canngbina* L. feeding on lipopolysaccharide-induced body weight loss and food intake in the zebrafish

**Fig 3:**
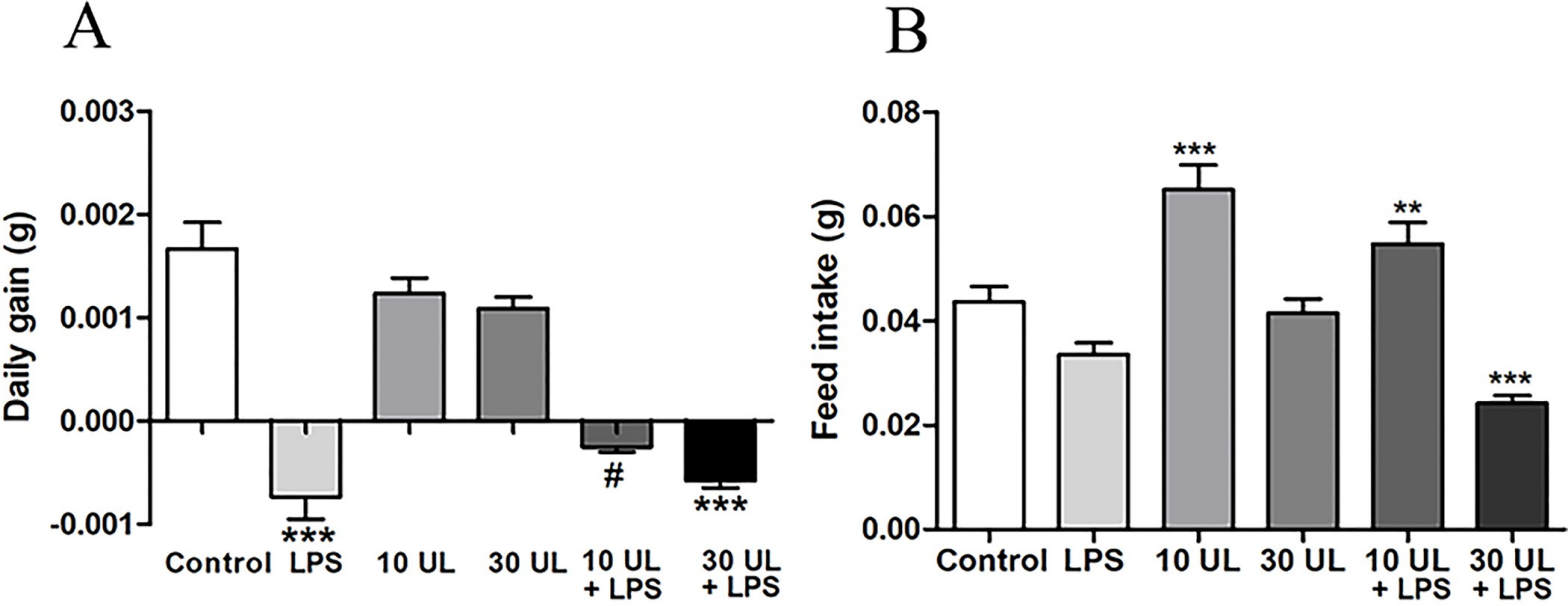
The inhibitory effect of *Urtica cannabina* L. (UL) feeding on lipopolysaccharide (LPS)-induced body weight loss and food intake in the zebrafish. (A): Changes in the body weight of the zebrafish in each group were detected after 30 d of feeding supplemented with UL and/or LPS. (B): Feed intake each day. The asterisks (*) indicate significant differences between the exposed and control groups (*p < 0.05; **p < 0.01; ***p < 0.001). The # indicates significant differences between the exposed and LPS groups (#: *P* < 0.05). Data are depicted as mean ± the standard deviations (n = 10).

The growth conditions of the control, 10 mg/g UL, and 30 mg/g UL treatment groups were similar, with no significant differences (Fig 3A). Compared with the control group, the daily weight gain of zebrafish in the LPS group decreased by nearly 70% (*p* < 0.001). Furthermore, the daily weight gain of zebrafish in the 10 and 30 mg/g UL+LPS groups decreased significantly, compared with the LPS group, and was 2.91 times (*p* < 0.001) and 1.27 times higher, respectively. Compared with the control group, LPS treatment inhibited the feed intake of zebrafish, but there was no significant difference (Fig 3B), whereas the feed intake of the 10 mg/g UL group and the 10 mg/g UL + LPS group increased 1.5 times (*p* < 0.001) and 1.25 times (*p* < 0.01) respectively. However, the feed intake of the 30 mg/g UL group did not change significantly compared with the control group, while that of the 30 mg/g UL +LPS group was significantly reduced (*p* < 0.001).

### Effects of *Urtica cannadina* L. feeding on lipopolysaccharide-induced inflammation in the zebrafish intestinal tissue

**Fig 4:**
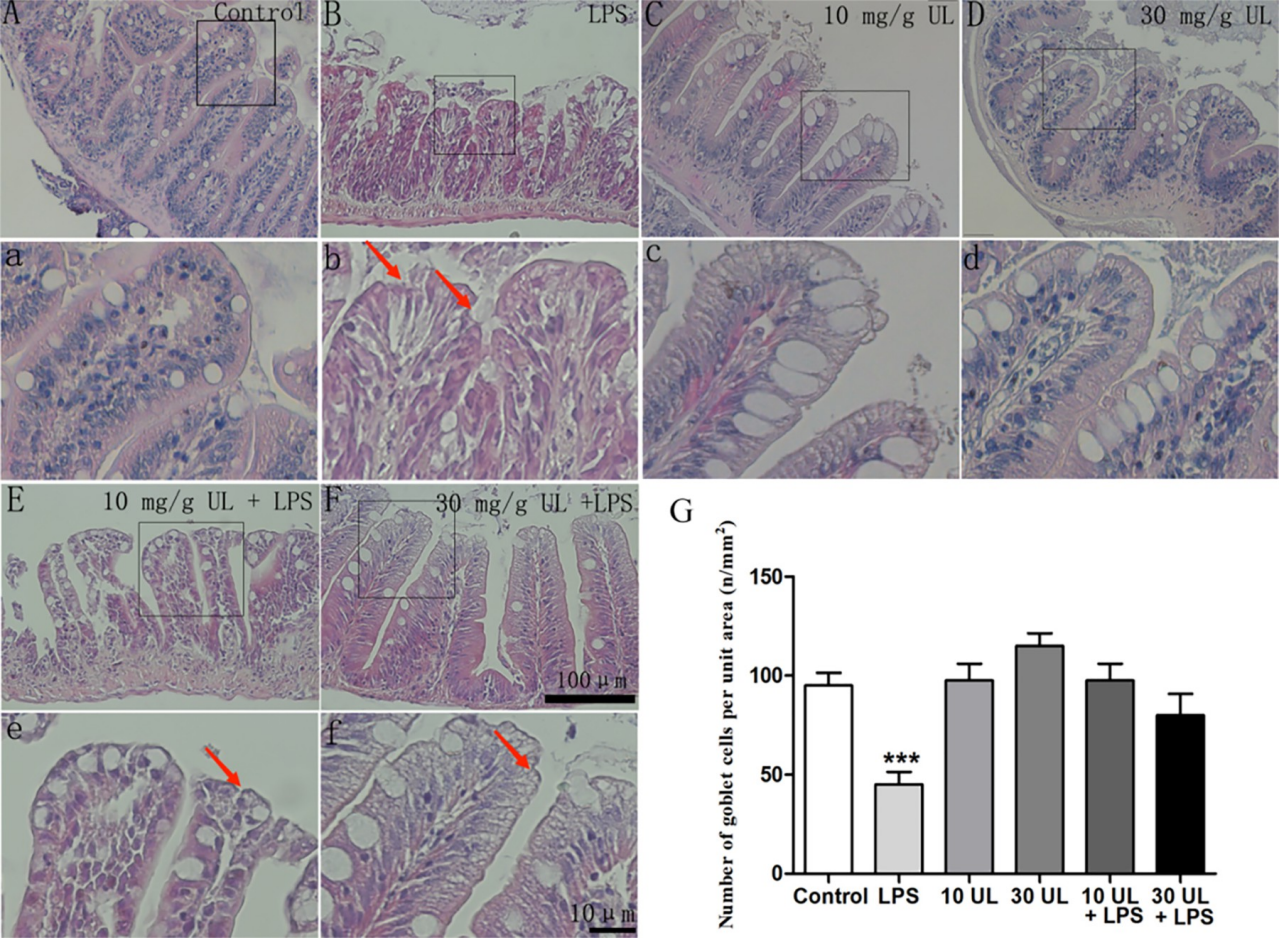
Inhibitory effect of *Urtica cannabina* L. (UL) feeding on the lipopolysaccharide (LPS)-induced reduction in the intestinal goblet cell numbers of the zebrafish. The zebrafish were treated with UL and LPS (LPS + UL) for 30 d. (A): The control group. (B): LPS exposure group. (C):10 mg/g UL feeding group. (D): 30 mg/g UL feeding group. (E): LPS + 10 mg/g UL treatment group. (F): LPS + 30 mg/g UL treatment group. (G): The number of intestinal goblet cells in each group. The asterisks (*) indicate significant differences between the exposed and control (*p < 0.05; **p < 0.01; ***p < 0.001). The data are presented as the mean ± the standard deviation (n = 10). Scale bar = 10 μm. The red arrows indicate structural damage to the villi.

The addition of the UL did not significantly change the intestine morphology (Fig 4A, 4C and 4D). Long and intact villi structures were observed with prominent goblet cells, and central blood capillaries. Furthermore, the microvilli were smooth, rounded, and had structural integrity. Moreover, the LPSs induced villi cells to become more eosinophilic (Fig 4B), and there were significantly fewer goblet cells (Fig 4G). The microvilli had a rough surface and showed signs of structural damage (Fig 4G, red arrows). In addition, the population of goblet cells in the LPS-exposed group was decreased by 49% (*p* < 0.001) compared to the control group (Fig 4A and 4B). Upon UL supplementation, the LPS-induced reduction of the goblet cells was alleviated, and the number of cells returned to the control levels (Fig 4E and 4F). However, the microvilli’s broken structure was not completely repaired (Fig 4E and 4F, red arrows).

### The effect of *Urtica cannabina* L. on lipopolysaccharide-induced inflammation-related gene expression in the zebrafish

**Fig 5:**
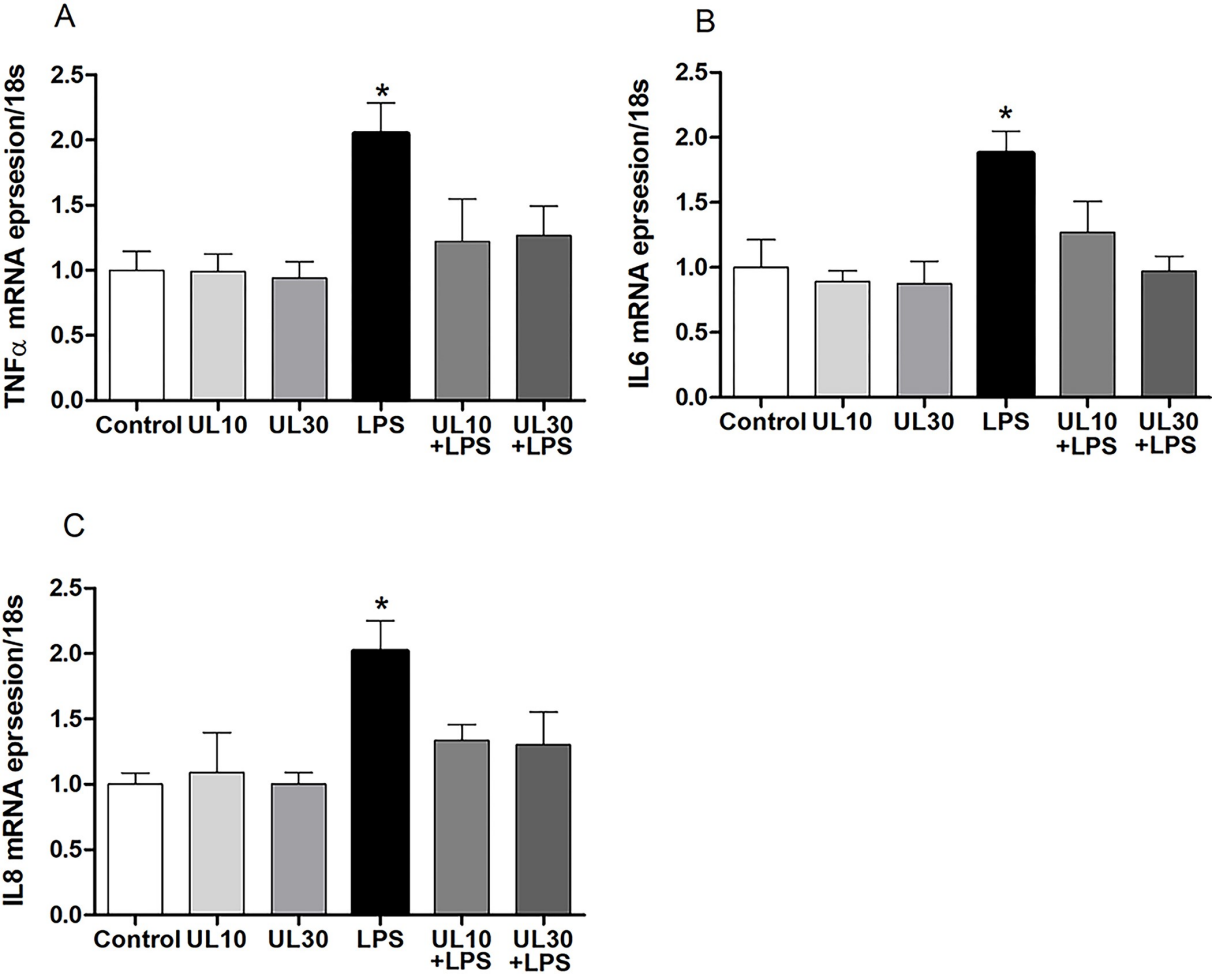
Effect of *Uritica cannabina* L. (UL) on lipopolysaccharide (LPS)-induced inflammation-related gene expression in the zebrafish. The mRNA expressions of (A): *TNF-α*, (B): *IL-6*, and (C): *IL-8*. The asterisks (*) indicate significant differences between the exposure group and control groups (*p < 0.05). The data are depicted as the mean ± the standard deviation (n = 5–6).

The expressions of the pro-inflammatory cytokines TNF-α, IL-6, and IL-8 in the liver were measured. When compared with the control group, the LPSs significantly increased the expression of *TNF-α*, *IL8*, and *IL-6* by 2.05, 2.01, and 1.87 times, respectively (*p* < 0.05; Fig 5). In both the UL treatments (10 and 30 mg/g), no significant changes were observed in the expression of the *TNF-α*, *IL-6*, *and IL-8* genes when compared with that of the control. However, the addition of UL rescued the effect of the LPSs on the expression of the three genes.

### Effects of *Urtica cannabina* L. feeding on the intestinal microflora of the zebrafish after lipopolysaccharide exposure

**Fig 6:**
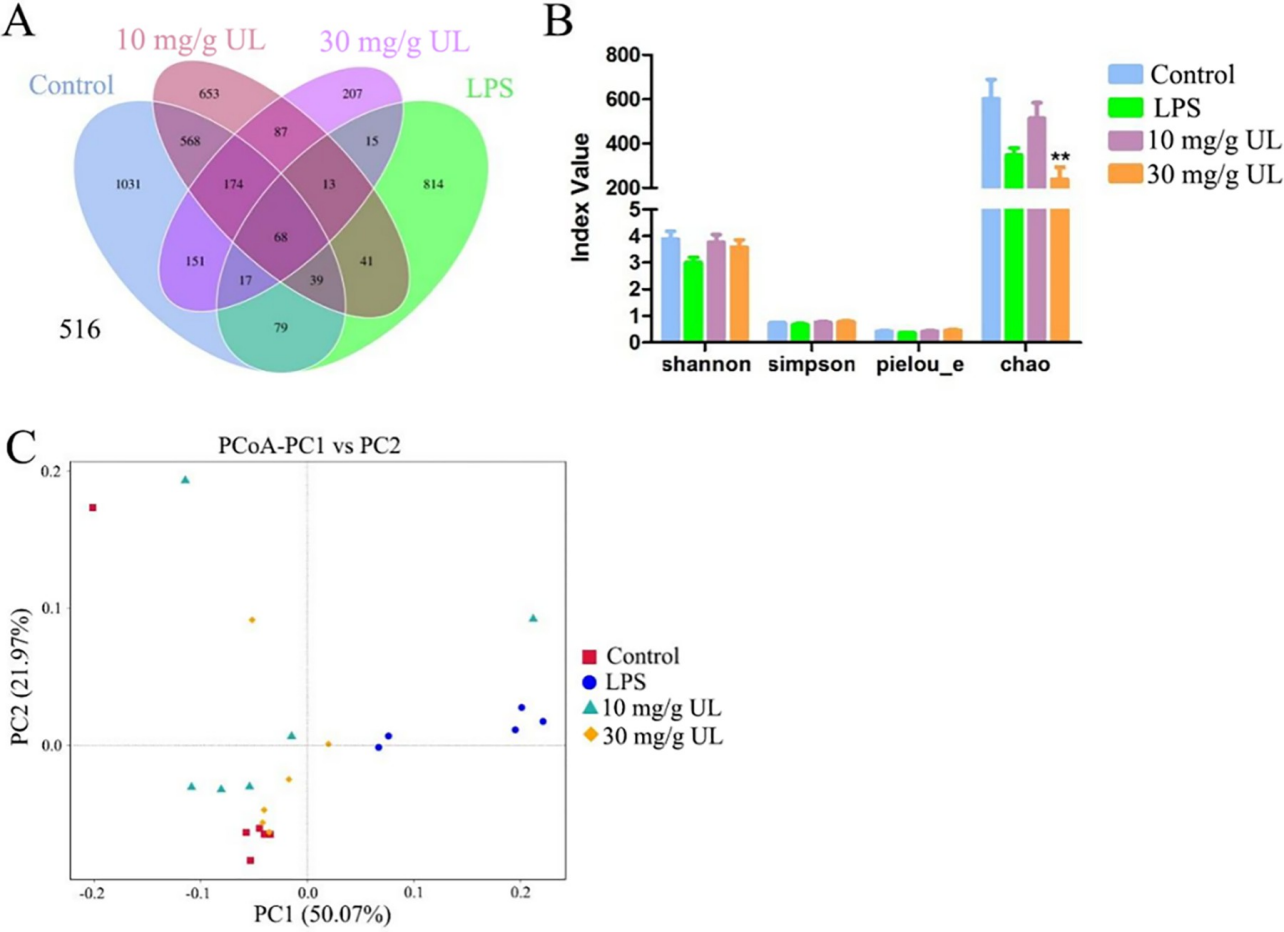
Effects of *Urtica cannabina* L. (UL) feeding on the microbial community of the adult zebrafish. (A): A Venn diagram showing the shared and distinct operational taxonomic unit numbers. (B): Microbial diversity index analysis and (C): Principal coordinate analysis (PCoA) of the gut microbiota of the control, lipopolysaccharide (LPS), 10 mg/g UL, and 30 mg/g UL zebrafish groups. The PCoA plots were generated using the operational taxonomic unit-level composition data. The asterisks (*) indicate significant differences between the exposed and control groups (*p < 0.05; **p < 0.01; ***p < 0.001). The data are illustrated as the mean ± the standard deviation (n = 10). Abbreviations: PC: Principal coordinate.

Based on the V3-V4 region of the 16S rRNA gene, a total of 3957 OTUs were identified from all the samples based on a threshold of 97% sequence similarity. Of these, about 68 OTUs were common taxa in all three treatments, while 1031, 653, 207, and 814 OTUs were unique to the control, low UL diet, high UL diet, and LPS groups, respectively. Furthermore, about 203, < 849, and 410 OTUs were shared between the LPS and the control groups, 10 mg/g UL and the control groups, and 30 mg/g UL and the control group, respectively (Fig 6A). (Login account: X101SC21114074-Z01-J002)

**Fig 7:**
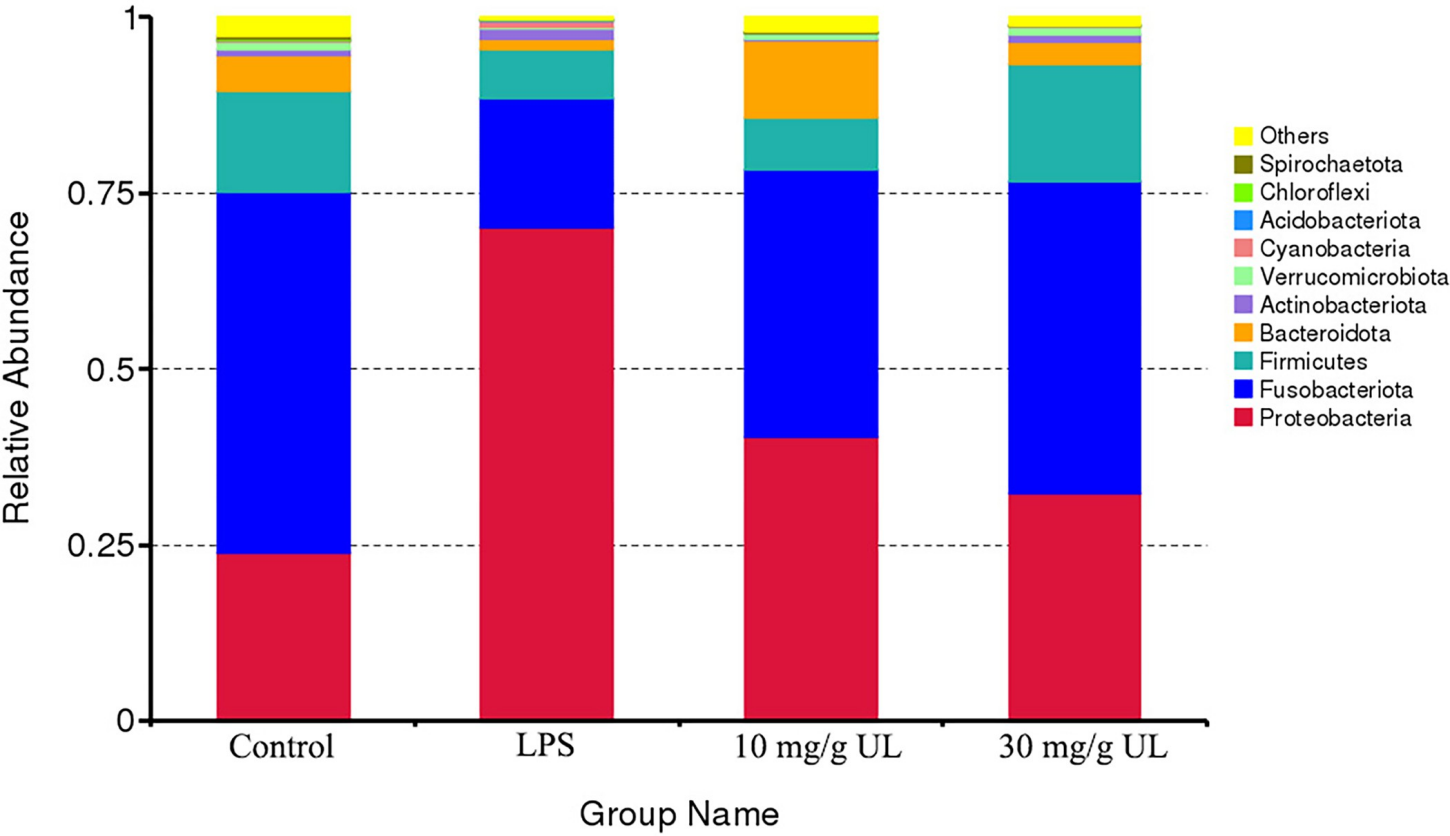
Community composition analysis of the gut microbiota of the adult zebrafish at the phylum level after 30 d of *Urtica cannabina* L. (UL) feeding.

The diversity indices showed a similar trend, where the Shannon and Simpson’s diversity indices had similar values for the three treatments; however, the Chao index showed that a high UL diet significantly reduced microbial diversity (Fig 6B). Furthermore, the principal component analysis (Fig 6C) indicated that a high UL diet promoted a unique gut microbiota composition, whereas small differences were observed between the low UL diet and control. Moreover, based on the microbial community composition, a phylum-level analysis was conducted (Fig 7). It was observed that compared with the control group, the relative abundance of the Proteobacteria in the intestinal flora of the zebrafish in the LPS group was significantly increased (*p* < 0.05), while that of the Fusobacteriota, Firmicutes, and Bacteroidetes decreased significantly. In addition, the relative abundance of the Firmicutes decreased, while that of the Bacteroidetes increased in the 10 mg/g UL group compared with the control group. In the 30 mg/g UL group, the relative abundance of the Firmicutes increased, and that of the Bacteroidetes decreased compared with the control group.

## Discussion

In recent years, many countries have legislated against the addition of antibiotics to feed, and researchers are searching for alternatives to antibiotics, such as immuno-suppressants, medicinal plants, nucleotides, organic acids, prebiotics, probiotics, and synthetic probiotics [32]. The studies on the use of medicinal plants as feed additives have a research hotspot, such as herbs, spices, seaweeds, herbal extracts, traditional Chinese medicine, and commercial plant derivatives. This is because medicinal plants as feed additives not only avoid environmental pollution but also enhance the immunity of the organisms. The literature has indicated comparable immune-induction and disease-resistance effects of medicinal plants in fish [32,33]. Many studies have investigated the efficacy of various plant products in aquafeeds, such as herbs [2], roots [34], and seed meal [35]. Furthermore, it has also been observed that medicinal plant extracts induce anti-inflammatory genes in fish at the genetic level, such as turmeric (*Curcuma longa*), spirulina (*Arthrospira platensis*), and sage (*Salvia officinalis*), trans-cinnamic acid from cinnamon, and *Urtica dioica* [36]. Among these plant extracts, nettles have a long history of use in traditional medicine. Zare *et al*. (2023). reported that fish fed with nettle had growth-promoting, immunity-enhancing, antiviral, antibacterial, and antiparasitic properties [37].

*Urtica cannabina* L. is widely distributed in Asia, Africa, and Europe, where it is used as food and herbal medicine [38]. The comparison of its various medicinal effects revealed that the anti-inflammatory effect of UL leaves was the most prominent [39,40]. Here, it was revealed that UL is a relatively safe plant feed with strong anti-inflammatory properties. Acute inflammation induced by tail cutting is a well-established model for the study of inflammation and regeneration in zebrafish. Tail-cutting experiments can be performed on zebrafish from the embryonic stage to maturity [40]. Caudal fin inflammation promotes the accumulation of the body’s immune cells at the wound site to eliminate inflammation and significantly increases TNF-α signaling [41]. He *et al*. reported that Cannabis leaf extract had a significant effect on the elimination of macrophages and neutrophils in the zebrafish’s caudal fin [42]. Moreover, propolis is composed of various organic compounds, such as phenolics and flavonoids, with antioxidant and anti-inflammatory activities, which can improve caudal fin regeneration [43]. These data support the results observed in the 10 mg/g UL group of this study, which might be related to organic compounds, such as the phenols and flavonoids of UL, which have good antioxidant and anti-inflammatory activities [44]. In addition, 30 mg/g UL had no significant effect on tail recovery; however, scars were observed, possibly due to the excessive antioxidant effect of UL.

This research found that LPSs decreased the body weight of the zebrafish, which was slowed down by UL administration. LPSs are the outer membrane component of gram-negative bacteria, and they stimulate inflammation and have appetite-suppressing properties [40]. Knopp *et al*. indicated that LPSs caused significant weight loss in adult mice (Mus musculus Linnaeus, 1758) [45], which supported its characteristics and is consistent with the present research results. Furthermore, Mehrabi *et al*. found that rainbow trout had enhanced growth performance and improved feed use efficiency when they were fed UL [29]. The LPS-induced reduction of zebrafish body weight might be related to the removal of the intestinal mucosa, the reduction of the goblet cells, and the impact on nutrient absorption. This effect was alleviated when UL was added.

Lipopolysaccharides are an endotoxin in the cell wall of gram-negative bacteria. Thus, LPS-induced inflammation in zebrafish is used as a model for anti-inflammatory drug research. Here, it was revealed that LPSs caused the decay and disappearance of the intestinal goblet cells and the weakening of the intestinal villi, whereas the UL supplementation in the diet effectively improved the number and size of the intestinal goblet cells, validated by Zhang *et al*. [17]. Similar findings suggested that the methanol extract of nettles (0.1 g/kg) promoted growth performance and improved metabolic and immune function in cichlid fish (Cichlidae) [46]. Dietary supplementation with UL can effectively protect the morphology and number of intestinal goblet cells as well as the intestinal villi morphology of the zebrafish, supported by the research of Guo *et al*. [47,48]. In addition, Lu *et al*. [49] suggested that the zebrafish toll-like receptor 4 (TLR4) can bind to LPSs, thereby activating the NF-κB signaling pathway. This research revealed that UL reduced the inhibitory effect of LPSs on the zebrafish intestinal goblet cells, which reached the same level as that of the control group. This data indicates that UL not only protects the intestinal goblet cells but also promotes intestinal digestion and absorption.

Inflammatory processes are mediated by macrophages, neutrophils, eosinophils, and mononuclear phagocytes [50]. Inflammatory immune responses are characterized by the expression of inflammatory mediators, such as TNF-α, and extensive immune cell infiltration [51]. Furthermore, anti-inflammatory processes can block the synthesis and release of inflammatory cytokines by inhibiting immune cell (mainly macrophages and neutrophils) aggregation at the inflammatory site [52]. When pro-inflammatory factors act on the organism, the immune system is activated and releases inflammatory cytokines, such as IL-6 and TNF-α, that stimulate an inflammatory response [52,53]. Therefore, LPSs can up-regulate reactive oxygen species (ROS) and the expression of inflammation-related factors, such as IL-1b, IL-6, and TNF-α in zebrafish [54]. Zhou *et al*. also induced intestinal inflammation in mice with LPSs and promoted abnormally high expression of inflammatory factors such as IL-6, IL-8, IL-10, and TNF-α [54]. In this study, LPSs abnormally increased the expression of the inflammatory factors IL-6, IL-8, and TNF-α in the zebrafish, while UL administration had the opposite response, suggesting that UL can effectively relieve LPS-induced intestinal inflammation. Therefore, some of the active substances in UL might reduce the abnormal expression of LPS-induced inflammation-related factors (such as IL-8, IL-6, and TNF-α) and restore them to normal levels by regulating ROS or the NF-κB pathway.

In the LPS-induced zebrafish, high and low UL concentrations had different effects on the intestinal microbiome. The LPS and control groups had the same microbiota number, where the 10 mg/g UL and control groups, as well as the 30 mg/g UL and control groups, shared fewer OTUs. The LPS group indicated the lowest intestinal microflora diversity and the correlation among the intestinal microflora was the lowest when compared with that of the other three groups. These data were consistent with the results of Liu *et al*. [55]. The intestinal flora of the zebrafish in the UL feeding group was similar to that in the control group. When compared with the control group, the relative abundance of the Proteobacteria in the LPS group significantly increased, while that of the Fusobacteriota, Firmicutes, and Bacteroidetes significantly decreased. Furthermore, in the 10 mg/g UL group, the relative abundance of the Firmicutes decreased, while that of the Bacteroidetes increased compared with the control group. This result was consistent with the data of Biao *et al*. [56], where there was an increase in the abundance of Bacteroidetes, and Danggui Shaoyao Powder had a strong effect on nonalcoholic fatty liver disease. This mechanism may be related to regulating the intestinal flora, which protects the intestinal mucosal barrier, regulates the TLR4 signaling pathway, and reduces the inflammatory response.

In this study, the relative abundance of the Firmicutes increased in the 30 mg/g UL group, while the relative abundance of the Bacteroidetes decreased. This was similar to a study that found that Lycium barbarum L. polysaccharides improved non-alcoholic fatty liver disease by increasing the relative abundance of Bacteroides and reducing the Proteus and Firmicutes/Bacteroides ratios, which inhibited liver inflammation and restored the intestinal microbiota as well as intestinal barrier [57]. In addition, two active tripeptides that were extracted from egg whites reduced jejunal inflammation and increased microbial community diversity in mice by significantly increasing the abundance of Bacteroides and decreasing the abundance of Firmicutes and Proteobacteria [58], consistent with this study. Although compared with the control group, the 10 and 30 mg/g UL groups indicated slightly increased relative abundance of Proteobacteria, the 10 mg/g UL dose enhanced the proportion of the dominant intestinal flora by increasing the relative abundance of Bacteroidetes to improve the anti-inflammatory ability in the intestinal tract. In addition, the 10 mg/g UL and control groups shared more flora. Moreover, the richness and diversity of the intestinal flora were much higher in the 10 mg/g UL group than in the LPS group, which provides a basis for UL preventing and inhibiting the invasion of potentially pathogenic microorganisms. Furthermore, these findings validate that UL is a superior plant feed additive that can replace antibiotics and enhance livestock immunity. Although these preliminary results support UL as an additive with anti-inflammatory effects and the ability to influence intestinal flora, UL’s limitations and advantages still need comprehensive research. However, this study supports the potential of UL as an additive for the health protection of livestock and poultry.

## Conclusions

In summary, UL has a protective effect against LPS-induced inflammatory changes in zebrafish. It reduced caudal fin scarring in the zebrafish and promoted its regeneration at a 10 mg/g dose, similar to that of the control group. Furthermore, UL suppressed LPS-induced weight loss and significantly alleviated intestinal goblet cell loss, as well as the number of intestinal goblet cells and intestinal villi in the zebrafish. Moreover, UL significantly inhibited LPS-induced expression of *TNF-α*, *IL-8*, and *IL-6*. This might be because UL increases the zebrafish intestinal probiotics, such as Bacteroidetes, and decreases pathogenic bacteria, such as Clostridium and Firmicutes. The findings showed that UL is a relatively safe plant feed with strong anti-inflammatory properties; thus, it could potentially be used as a growth and anti-inflammatory additive for livestock.
