# Correction: Anti-inflammatory effects and beneficial effects of the feed additive *Urtica cannabina* L. in zebrafish

**DOI:** 10.1371/journal.pone.0322713

**Published:** 2025-04-10

**Authors:** The PLOS One Staff

There are errors in the author affiliations. The correct affiliations are as follows:

Wuyun Liu^1,2,3^, Huarong Yu^1^, D. Gurbazar^2^, D. Rinchindorj^2^, Wei Kang^3^, Chelimuge Qi^3^, Hongsong Chen^3^, Xu Chang^3^, Huan You^4^, Yongmei Han^3^, Zhigang Li^1^, Ahmed R. G.^5^, Wu Dong^3^

1 Key Laboratory of Ecological Agriculture in Horqin Sandy Land, State Ethnic Affairs Commission, China, College of Agriculture, Inner Mongolia Minzu University, Tongliao, Inner Mongolia, China, 2 Mongolian University of Life Sciences, School of Animal Science & Biotechnology, Ulaanbaatar, Mongolia, 3 Inner Mongolia Key Laboratory of Toxicant Monitoring and Toxicology, College of Animal Science and Technology, Inner Mongolia Minzu University, Tongliao, Inner Mongolia, China, 4 Tongliao Animal Husbandry Development Center, Tongliao, Inner Mongolia, China, 5 Faculty of Science, Zoology Department, Division of Anatomy and Embryology, Beni-Suef University, Beni-Suef, Egypt.

The publisher apologizes for the error.

## References

[1] Liu W, Yu H, Gurbazar D, Rinchindorj D, Kang W, Qi C, et al. Anti-inflammatory effects and beneficial effects of the feed additive *Urtica cannabina* L. in zebrafish. PLoS One. 2024;19(7): e0307269. doi: 10.1371/journal.pone.0307269 39018284 PMC11253947

